# Supplementary material for: Mechanisms governing the pioneering and redistribution capabilities of the non-classical pioneer PU.1
Source: Nat Commun. 2020 Jan 21;11:402. doi: 10.1038/s41467-019-13960-2 (PMC6972792; doi:10.1038/s41467-019-13960-2)
Supplement: Supplementary file 7 — Source data [file 41467_2019_13960_MOESM7_ESM.zip › Source_Data/FigureS2/FigureS2B_MotifScanOutput/KmeansCluster2/homerResults/motif19.similar.html]

motif19

## Information for motif19

C
A
G
T
T
A
C
G
G
A
T
C
C
G
A
T
A
T
G
C
A
G
T
C
A
G
C
T
A
G
T
C
G
T
A
C
T
G
A
C
  
Reverse Opposite:  

A
C
T
G
C
A
T
G
T
C
A
G
T
C
G
A
T
C
A
G
T
A
C
G
C
G
T
A
C
T
A
G
A
T
G
C
G
T
C
A
  

|  |  |
| --- | --- |
| p-value: | 1e-59 |
| log p-value: | -1.367e+02 |
| Information Content per bp: | 1.672 |
| Number of Target Sequences with motif | 390.0 |
| Percentage of Target Sequences with motif | 7.76% |
| Number of Background Sequences with motif | 1127.2 |
| Percentage of Background Sequences with motif | 2.77% |
| Average Position of motif in Targets | 102.8 +/- 49.9bp |
| Average Position of motif in Background | 101.3 +/- 77.6bp |
| Strand Bias (log2 ratio + to - strand density) | 0.0 |
| Multiplicity (# of sites on avg that occur together) | 1.06 |
| Motif File: | file (matrix) reverse opposite |

### Similar de novo motifs found

|  |  |  |  |  |  |  |  |
| --- | --- | --- | --- | --- | --- | --- | --- |
| Rank | Match Score | Redundant Motif | P-value | log P-value | % of Targets | % of Background | Motif file |
| 1 | 0.845 | T C G A C T A G A C T G C T A G T C G A A T C G A C T G C G T A C T A G | 1e-47 | -110.344887 | 19.95% | 12.17% | motif file (matrix) |
